# Site-Specific Labeling of Proteins with Near-IR Heptamethine Cyanine Dyes

**DOI:** 10.3390/molecules23112900

**Published:** 2018-11-07

**Authors:** Chen-Ming Lin, Syed Muhammad Usama, Kevin Burgess

**Affiliations:** Department of Chemistry, Texas A & M University, Box 30012, College Station, TX 77842, USA; chen-ming_lin@tamu.edu (C.-M.L.); syed.usama@chem.tamu.edu (S.M.U.)

**Keywords:** heptamethine cyanine, protein labeling, thiol labeling, cancer targeting, vimentin

## Abstract

Convenient labeling of proteins is important for observing its function under physiological conditions. In tissues particularly, heptamethine cyanine dyes (Cy-7) are valuable because they absorb in the near-infrared (NIR) region (750–900 nm) where light penetration is maximal. In this work, we found Cy-7 dyes with a *meso*-Cl functionality covalently binding to proteins with free Cys residues under physiological conditions (aqueous environments, at near neutral pH, and 37 °C). It transpired that the *meso*-Cl of the dye was displaced by free thiols in protein, while nucleophilic side-chains from amino acids like Tyr, Lys, and Ser did not react. This finding shows a new possibility for convenient and selective labeling of proteins with NIR fluorescent probes.

## 1. Introduction

**Figure 1:**
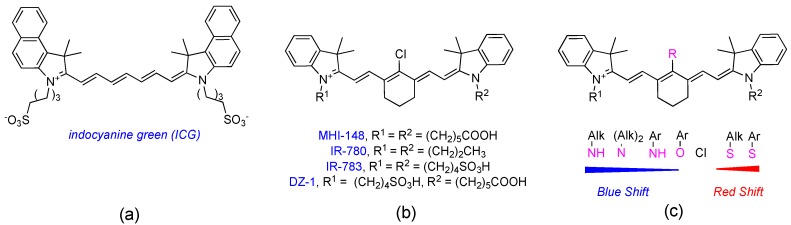
Structures of: (**a**) ICG; (**b**) cancer-tissue-targeting Cy-7 dyes and their *meso*-substituted derivatives; and (**c**) graphical representation of the effects of *meso*-substitution on electronic spectra.

Hydrophilic near-infrared (NIR) fluorescent dyes are valued for in-depth imaging in tissues, and heptamethine cyanines, or Cy-7 dyes, which absorb in the NIR region (700–900 nm), are amongst the most widely used [1]. Indocyanine green (ICG, Figure 1), the only FDA-approved Cy-7 dye, has been widely used in medical and clinical diagnostics [2,3,4]. 

Many applications of Cy-7 dyes require that they be covalently conjugated to, for example, antibodies, cell surface targeting peptides/biomarkers, and small molecule substrates. This is often achieved by modifying Cy-7 derivatives with coupling functionalities such as maleimide, succinimide esters, isocyanates, or sulfonyl halides. The challenge with strategies like this is balancing the demands of experimental convenience with selectivity towards targeted amino acid types. Extensive modifications to Cy-7 dyes can also alter their solubility and photophysical properties [5]. 

Figure 1 shows dyes featured in this study. Probes of this type, i.e., with a 1-chloro-2,6-disubstituted cyclohexane (i.e., MHI-148, IR-780, IR-783, and DZ-1) [6,7,8], are known for their tumor localizing properties [9,10,11,12]. Therefore, these Cy-7 dyes are potential carriers of cytotoxic payload for combined cancer targeted therapy and imaging [13,14,15,16]. In our research in this area, we happened to make a surprising finding regarding such a conjugation process. Specifically, when investigating in vitro reactions and cell lysates featuring MHI-148, it was found to covalently bind to several proteins with high selectivity as evidenced by gel electrophoresis and NIR imaging at around 800 nm.

We hypothesized that the *meso*-Cl of MHI-148 was substituted by nucleophilic functional groups of amino acids (Cys, Ser, Tyr and Lys) of proteins. This paper provides data to support this hypothesis and understand the selectivity. 

## 2. Results and Discussion

### 2.1. Syntheses of Amino-Acid-Substituted Cy-7 Dyes

**Scheme 1:**
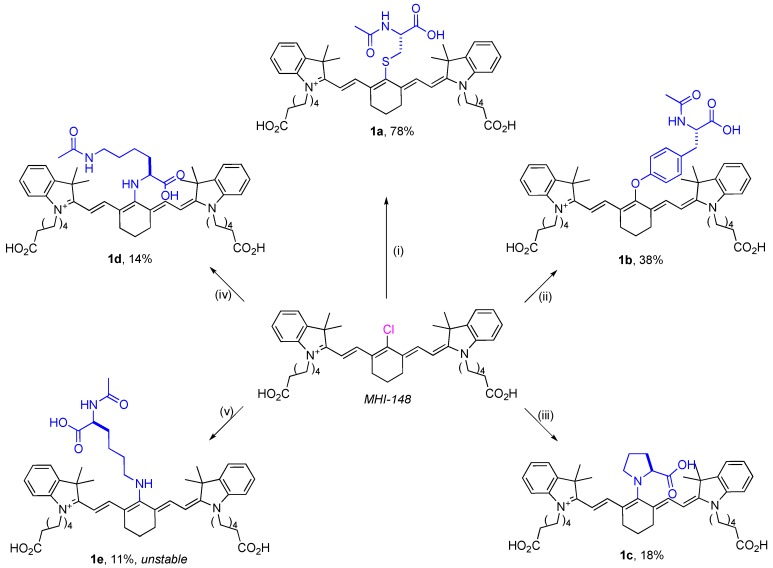
Preparation of amino-acid-substituted Cy-7 dyes. (i) *N*-acetyl-L-cysteine (1 eq.), ^i^Pr_2_NEt (1.5 eq.), DMF, 25 °C, 1 h (ii) *N*-acetyl-L-tyrosine (1 eq.), NaH, DMF, 25 °C, 18 h (iii) proline (1 eq.), ^i^Pr_2_NEt (1 eq.), DMF, 60 °C, 2 h (iv) *Nε*-acetyl-L-lysine (1 eq.), ^i^Pr_2_NEt (1 eq.), DMF/H_2_O, 60 °C, 20 h (v) *N*α-acetyl-L-lysine (1 eq.), ^i^Pr_2_NEt (1 eq.), DMF, 60 °C, 20 h.

Reactions under controlled conditions were used to test if nucleophilic substitution of the chloride of MHI-148 possibly occurred in DMF solvent. Thus, several amino acids with different nucleophilic side-chains (*N*-acetyl-l-cysteine, *N*-acetyl-l-tyrosine, *Nα*-acetyl-l-lysine, *Nε*-acetyl-l-lysine, and l-proline) were reacted with MHI-148 under conditions that were varied to force the reactions to proceed. The corresponding amino-acid-substituted Cy-7 dyes were indeed formed (Scheme 1); these were isolated, characterized (see supplementary materials for NMR and mass spectrometry), and later used as standards for comparison of high-performance liquid chromatography (HPLC) retention times. Serine was excluded from these experiments because alcohol hydroxyl groups are known to not substitute the *meso*-Cl without complications [17,18,19,20]. 

*N*-Acetyl-L-cysteine was the most reactive nucleophile of the five amino acids studied. In the presence of Hünig’s base, MHI-148 was completely converted to the thiol-substituted product within 1 h at 25 °C in DMF (0.1 M 1:1 dye:Cys-derivative, LC-MS analyses). Four other amino acid nucleophiles studied required a stronger base (Tyr) and/or elevated temperatures (Pro and Lys), and even then, a significant amount of unreacted MHI-148 was observed after several hours. *N*α-Acetyl-L-lysine reacted faster than those three, giving the substituted product **1e**, which can be isolated by reversed-phase flash chromatography; however, significant decomposition was observed (color change from blue to pink) in organic solvents after only 20 min. The instability caused by delocalization of a lone pair of elections by primary amine at *meso* position is also reported by other groups [21,22]. Overall, this data indicated the *meso*-Cl substitution reactivity was thiol > phenol > 2° amine > 1° amine, which corresponds to the observations in the literature [23]. At this stage, we did not know if these reactivities would also be observed in aqueous media, but preferential conjugation of MHI-148 to Cys residues seemed more likely.

### 2.2. Optical Properties of Amino-Acid-Substituted Cy-7 Dyes

**Figure 2:**
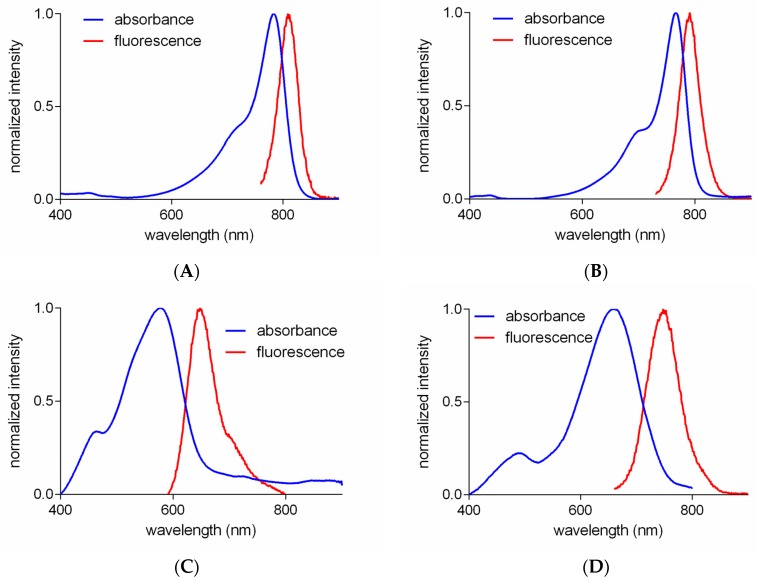
Normalized absorbance and fluorescence of compounds **1a**–**d** (6 µM, 37 °C) in pH 7.24 10 mM PBS buffer. (**A**) compound **1a**: λ_max abs_ 783 nm (blue), λ_max emiss_ 809 nm (red). (**B**) compound **1b**: λ_max abs_ 766 nm (blue), λ_max emiss_ 791 nm (red). (**C**) compound **1c**: λ_max abs_ 578 nm (blue), λ_max emiss_ 648 nm (red). (**D**) compound **1d**: λ_max abs_ 658 nm (blue), λ_max emiss_ 748 nm (red).

Figure 2 shows absorbance and fluorescence of compounds **1a**–**d** in 10 mM PBS buffer. The *S*- or *O*-substituted compounds (**1a** and **1b**) had absorbance and fluorescence spectra similar to those of MHI-148. Significant red-shifts of absorbance and fluorescence were observed for both *N*-substituted Cy-7 dyes (**1c** and **1d**), which have been attributed [24,25] to conjugation of the nitrogen lone pair with the Cy-7 core. Interestingly, the peaks for the *N*-substituted products are significantly broader, implying more vibrational fine structures than the *S*- or *O*-substituted products [26].

### 2.3. Meso-Cl Functionality of Cy-7 Dyes Is Essential for Cys-Selective Protein Labeling 

**Figure 3:**
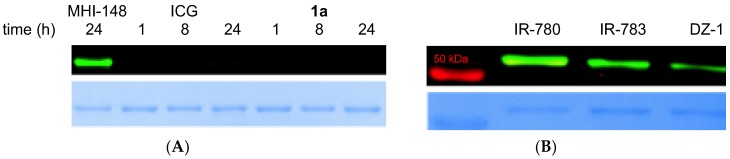
Near-infrared (NIR) fluorescent gel image of (**A**) vimentin (1 μM) incubated with different cyanines (10 μM) in 50 mM pH 7.24 HEPES buffer at different incubation times; (**B**) vimentin (1 μM) incubated with IR-780, IR-783, and DZ-1 in the same buffer as (**A**) for 24 h. CBB-G250 staining indicated an equal amount of protein (100 ng) was loaded into gel.

Vimentin, a structural protein, was chosen for study because it has only one Cys residue (C328). Vimentin (1 μg, 1 μM) was incubated with Cy-7 dyes containing *meso*-Cl (MHI-148, IR-780, IR-783, and DZ-1; 10 μM) and with Cy-7 dyes without *meso*-Cl (ICG and **1a**) for comparison (throughout, 10 μM in 50 mM pH 7.24 HEPES buffer at 37 °C for up to 24 h). An equal amount of the samples (100 ng) was electrophoresed under reducing conditions, and the gel was analyzed using an NIR imager. Only the Cy-7 dyes containing *meso*-Cl reacted to give a band observable at 800 nm (Figure 3). 

**Scheme 2:**
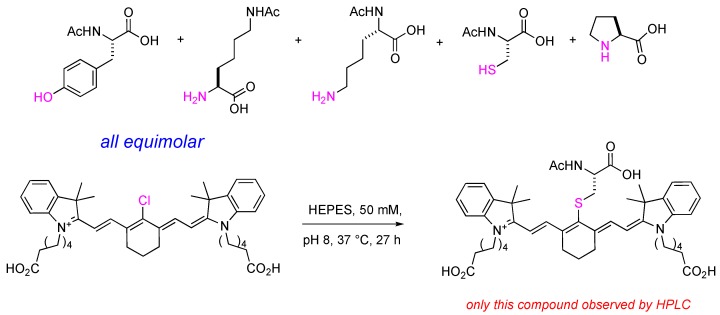
Competition study of MHI-148 with amino acids in aqueous buffer.

**Figure 4:**
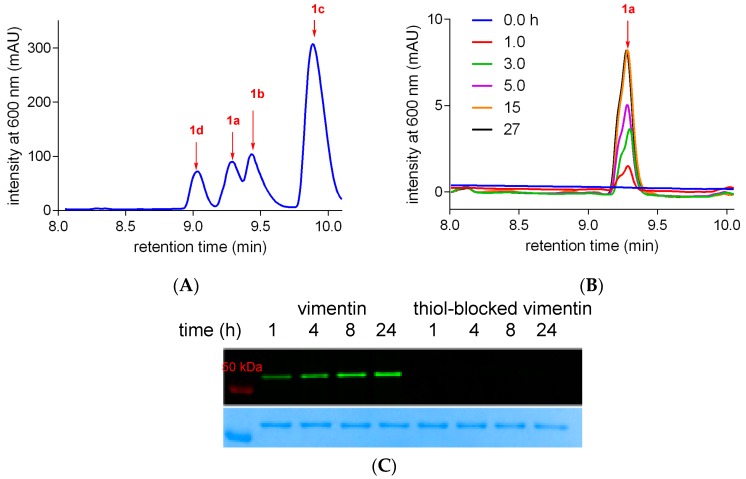
High-performance liquid chromatography (HPLC) analysis of (**A**) 200 μM of each amino-acid-conjugate standard **1a**–**d** in 50 mM pH 8.0 HEPES buffer; (**B**) kinetic study for 200 μM of MHI-148 with 200 μM of each amino acid (*N*-acetyl-L-Cys, *N*-acetyl-L-Tyr, *N*α-acetyl-L-Lys, *N*ε-acetyl-L-Lys, and L-proline) in 50 mM pH 8.0 HEPES buffer incubating at 37 °C; (**C**) NIR fluorescent gel image of vimentin or 6-MA-blocked vimentin (1 μM) incubated with MHI-148 (1 μM) in 50 mM pH 7.24 HEPES buffer at different incubation times.

Further evidence for the superior reactivities of Cys side-chains over other nucleophilic amino acid residues was obtained via competition experiments. Thus, MHI-148 (200 μM) in 50 mM pH 8.0 HEPES buffer was incubated with equimolecular amounts of five amino acids (Scheme 2) at 37 °C, and the reaction was monitored by HPLC up to 27 h. Prototypes of this experiment were intended to measure relative rates, however, only formation of the Cys-product **1a** occurred (HPLC spike with the standard from Scheme 1 (Figure 4) and LC-MS analyses). Under these conditions, approximately 32% of MHI-148 was substituted and 68% remained after 15 h. The reaction did not go to completion due to equilibrium of the reaction or due to oxidation of cysteines in aqueous conditions. This observation implied amine, alcohol, and phenol side-chains in the protein did not react with the dye.

Blocking experiments were performed to be absolutely sure that the vimentin Cys was the reactive group for coupling to MHI-148 in aqueous buffer at 37 °C. Thus, maleimide-blocked protein [27,28] was formed by incubating vimentin with 6-maleimidohexanoic acid (6-MA; 18 h in 50 mM pH 7.24 HEPES buffer at 37 °C). Vimentin and the thiol-blocked vimentin were incubated with MHI-148 for different incubation times, then analyzed using SDS-PAGE gel electrophoresis. Figure 4C shows that the concentration of MHI-148 covalently bound to vimentin progressively increased (NIR fluorescence at ~800 nm), whereas no fluorescent band was observed for the thiol-blocked vimentin. 

**Figure 5:**
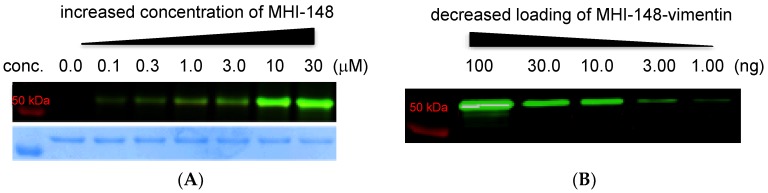
NIR fluorescent gel image of (**A**) vimentin (1 μM) incubated with different concentrations of MHI-148 in 50 mM pH 7.24 HEPES buffer for 3 h at 37 °C (**B**) 10:1 concentration ratio of MHI-148:vimentin sample was loaded into 10% SDS-PAGE gel with different amounts of vimentin sample.

Overall, based on all the experiments above, we concluded that MHI-148 selectively binds the only free Cys in vimentin, C328, in aqueous buffer at 37 °C, and went on to calibrate the efficiency of binding. Thus, NIR fluorescence (>800 nm) in gel electrophoresis was quantitative for vimentin (1 μM in 50 mM pH 7.24 HEPES buffer) when incubated with of MHI-148 (0–30 μM, 3 h at 37 °C). Fluorescence intensities of the salient band saturated at 10 μM (Figure 5a), which means the tested fluorescent compound can quantitatively label the protein at a 10:1 ratio within 3 h when incubated under these conditions in HEPES aqueous buffer. Experiments to test sensitivity revealed labeled vimentin was detectable at concentrations as low as 1 ng on our gel imaging apparatus (Figure 5b).

### 2.4. Labeling of Other Proteins Using MHI-148

**Figure 6:**
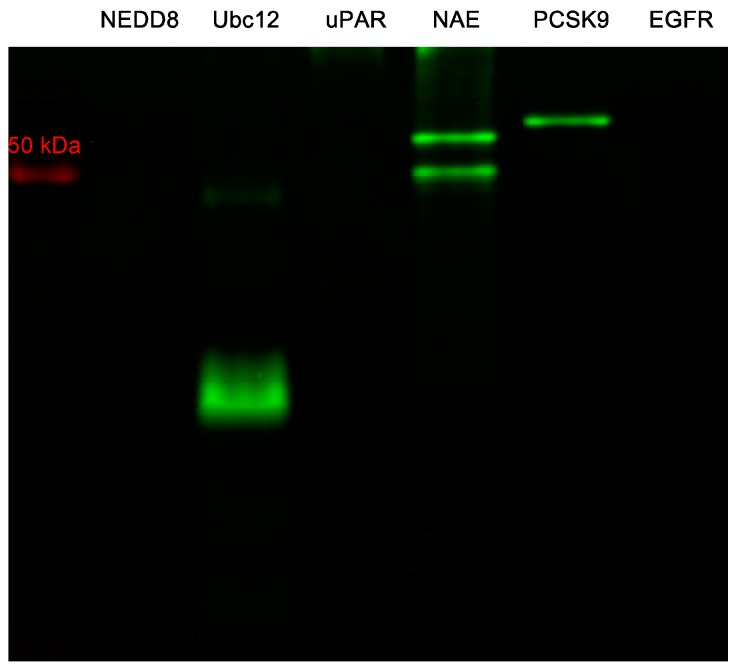
NIR fluorescent gel image of diverse proteins (4 μM) incubated with MHI-148 (4 μM) for 3 h using 50 mM pH 7.24 HEPES buffer at 37 °C.

Several proteins with and without free Cys residues were labeled to test the robustness of the method developed for vimentin. NEDD8-activating enzyme (NAE) [29], Ubc12 [30], and PCSK9 [31] contain free thiols (reduced Cys residues), whereas NEDD8 [29] (no Cys in sequences), truncated suPAR (residues 1-281, 12 disulfides) [32], and EGFR (25 disulfides) [33] have none. Figure 6 shows that only the proteins containing sulfhydryl groups reacted under the standard conditions. NAE consists of two subunits (APPBP1 and UBA3) which each contain free Cys; hence, two NIR fluorescence bands were observed for that sample. 

## 3. Materials and Methods 

### 3.1. General Information

All reactions were carried out with dry solvents under anhydrous conditions under an inert atmosphere (argon). Glassware was dried in an oven at 140 °C for a minimum of 6 h prior to use for all reactions. IR-783 and IR-780 were purchased from Sigma Aldrich (Atlanta, GA, USA) and abcr GmBH (Karlsruhe, Germany), respectively, and DZ-1 and MHI-148 were synthesized according to literature protocol [7,12,14,34]. All other reagents were purchased at a high commercial quality (typically 97% or higher) and used without further purification, unless otherwise stated. Products were purified using a reverse-phase column on a preparative high-performance liquid chromatography (prep HPLC) (Agilent, Santa Clara, CA, USA) obtained from solid-phase synthesis in 10%–95% MeCN/water with 0.05% trifluoroacetic acid over 20 min. High-field NMR spectra were recorded with Bruker Avance III (Billerica, MA, USA) at 400 MHz for ^1^H, and 100 MHz for ^13^C for all compounds. All spectra were calibrated using residual nondeuterated solvent as an internal reference (MeOD-*d*_4_: ^1^H-NMR = 3.30, ^13^C-NMR = 49.0, DMSO-*d*_6_: ^1^H-NMR = 2.50, ^13^C-NMR = 39.5). The following abbreviations were used to explain the multiplicities: s = singlet, d = doublet, t = triplet, q = quartet, quint = quintet, dd = double doublet, dt = double triplet, dq = double quartet, and m = multiplet. Electrospray ionization mass spectrometry (ESI-MS) data were collected on a triple-stage quadrupole instrument (Thermo Scientific, Waltham, MA, USA) in a positive mode. All statistical analyses were carried out by GraphPad Prism version 6.0 (GraphPad Software, La Jolla, CA, USA). 

### 3.2. Synthesis and Characterization

2-((E.)-2-((E)-2-(((R)-2-acetamido-2-carboxyethyl)thio)-3-(2-((E)-1-(5-carboxypentyl)-3,3-dimethylindolin-2-ylidene)ethylidene)cyclohex-1-en-1-yl)vinyl)-1-(5-carboxypentyl)-3,3-dimethyl-3H-indol-1-ium (**1a**)

To a solution of MHI-148 (25.0 mg, 0.04 mmol) in DMF (1.00 mL), *N*-acetyl-L-cysteine (5.98 mg, 0.04 mmol) and ^i^Pr_2_NEt (9.41 μL, 0.06 mmol) were added and the reaction was stirred at 25 °C for 1 h. Solvent was removed under a stream of nitrogen gas and purified by preparative reversed-phase HPLC (10%–95% CH_3_CN/water containing 0.05% TFA). Compound was lyophilized to obtain green solid (23.4 mg, 78%). ^1^H-NMR (400 MHz, MeOD) δ 8.77 (d, *J* = 13.9 Hz, 2H), 7.49 (d, *J* = 7.3 Hz, 2H), 7.45–7.36 (m, 2H), 7.27 (dd, *J* = 14.9, 7.6 Hz, 4H), 6.28 (d, *J* = 13.8 Hz, 2H), 4.55 (dd, *J* = 7.5, 5.3 Hz, 1H), 4.15 (t, *J* = 6.9 Hz, 4H), 3.40 (dd, *J* = 13.4, 5.3 Hz, 1H), 3.12 (dd, *J* = 13.4, 7.5 Hz, 1H), 2.75–2.54 (m, 4H), 2.31 (t, *J* = 7.3 Hz, 4H), 1.98–1.92 (m, 2H), 1.95 (s, 3H), 1.90–1.80 (m, 4H), 1.74 (s, 12H), 1.72–1.64 (m, 4H), 1.55–1.45 (m, 4H). ^13^C-NMR (100 MHz, MeOD) δ 177.23, 173.86, 173.21, 172.93, 157.83, 146.46, 143.72, 142.48, 134.54, 129.83, 126.27, 123.46, 112.00, 102.20, 54.36, 50.51, 50.49, 49.85, 44.97, 39.51, 34.61, 28.44, 28.02, 27.38, 25.65, 22.75, 22.03. HRMS calculated for C_47_H_60_N_3_O_7_S^+^ (M)^+^: 810.4146; found 810.4166.

6-((E.)-2-((E)-2-(2-(4-(2-acetamido-2-carboxyethyl)phenoxy)-3-((E)-2-(1-(5-carboxypentyl)-3,3-dimethyl-3H-indol-1-ium-2-yl)vinyl)cyclohex-2-en-1-ylidene)ethylidene)-3,3-dimethylindolin-1-yl)hexanoate (**1b**)

NaH (0.89 mg, 0.04 mmol) was added to a solution of *N*-acetyl-L-tyrosine (8.25 mg, 0.04 mmol) in DMF (1.00 mL) and the reaction was stirred at 25 °C for 30 min. MHI-148 (25.0 mg, 0.04 mmol) was then added to the above reaction and the reaction was stirred for an additional 18 h at 25 °C. Solvent was removed under a stream of nitrogen gas and purified by preparative reversed-phase HPLC (10%–95% CH_3_CN/water containing 0.05% TFA). Compound was lyophilized to obtain green solid (12.2 mg, 38%). ^1^H-NMR (400 MHz, MeOD) δ 8.01–7.92 (m, 2H), 7.36 (t, *J* = 7.8 Hz, 4H), 7.31–7.15 (m, 6H), 7.05 (d, *J* = 8.7 Hz, 2H), 6.13 (d, *J* = 14.2 Hz, 2H), 4.54 (dd, *J* = 9.1, 5.2 Hz, 1H), 4.09 (t, *J* = 7.3 Hz, 4H), 3.14 (dd, *J* = 14.0, 5.2 Hz, 1H), 2.84 (dd, *J* = 14.2, 9.2 Hz, 1H), 2.73 (t, *J* = 5.8 Hz, 4H), 2.30 (t, *J* = 7.3 Hz, 4H), 2.04 (t, *J* = 7.3 Hz, 2H), 1.84 (s, 3H), 1.83–1.73 (m, 4H), 1.67 (dd, *J* = 15.1, 7.5 Hz, 4H), 1.46 (t, *J* = 7.7 Hz, 4H), 1.33 (s, 12H). ^13^C-NMR (100 MHz, MeOD) δ 177.20, 174.59, 173.75, 173.05, 165.43, 160.25, 143.57, 143.34, 142.51, 132.96, 132.16, 129.76, 126.19, 123.40, 123.20, 115.75, 111.94, 100.93, 55.48, 50.27, 44.85, 37.62, 34.59, 28.25, 27.96, 27.33, 25.63, 25.21, 22.48, 22.39. HRMS calculated for C_53_H_64_N_3_O_8_^+^ (M)^+^: 870.4688; found 870.4675.

1-(5-C.arboxypentyl)-2-((E)-2-((E)-3-(2-((E)-1-(5-carboxypentyl)-3,3-dimethylindolin-2-ylidene) ethylidene)-2-((S)-2-carboxypyrrolidin-1-yl)cyclohex-1-en-1-yl)vinyl)-3,3-dimethyl-3H-indol-1-ium (**1c**)

To a solution of MHI-148 (25.0 mg, 0.04 mmol) in DMF (1.00 mL), L-proline (4.26 mg, 0.04 mmol) and ^i^Pr_2_NEt (6.27 μL, 0.04 mmol) were added and the reaction was stirred at 60 °C monitored by Agilent LC-MS. The reaction reached equilibrium after 2 h and the solvent was removed under a stream of nitrogen gas and purified by preparative reversed-phase HPLC (10%–95% CH_3_CN/water containing 0.05% TFA). Compound was lyophilized to obtain blue solid (5.18 mg, 18%). ^1^H-NMR (400 MHz, DMSO) δ 7.42 (d, *J* = 7.3 Hz, 2H), 7.28 (t, *J* = 7.7 Hz, 2H), 7.11 (d, *J* = 7.7 Hz, 2H), 7.04 (t, *J* = 7.2 Hz, 2H), 5.68 (d, *J* = 12.4 Hz, 2H), 4.96 (d, *J* = 7.3 Hz, 2H), 4.02–3.97 (m, 1H), 3.90–3.87 (m, 4H), 2.76–2.57 (m, 3H), 2.46–2.29 (m, 3H), 2.28–2.18 (m, 1H), 2.20 (t, *J* = 7.3 Hz, 4H), 2.09–1.98 (m, 3H), 1.79–1.70 (m, 2H), 1.70–1.62 (m, 4H), 1.57 (s, 6H), 1.61–1.48 (m, 4H), 1.54 (s, 6H), 1.45–1.30 (m, 4H). ^13^C-NMR (100 MHz, DMSO) δ 174.29, 172.71, 166.02, 158.00, 142.99, 139.74, 136.52, 128.11, 123.13, 122.28, 122.00, 109.00, 94.22, 64.69, 56.49, 46.93, 42.26, 33.52, 30.04, 29.59, 28.65, 28.11, 26.70, 25.81, 24.22, 20.65. HRMS calculated for C_47_H_60_N_3_O_6_^+^ (M)^+^: 762.4477; found 762.4457.

2-((E.)-2-((E)-2-(((S)-5-acetamido-1-carboxypentyl)amino)-3-(2-((E)-1-(5-carboxypentyl)-3,3-dimethylindolin-2-ylidene)ethylidene)cyclohex-1-en-1-yl)vinyl)-1-(5-carboxypentyl)-3,3-dimethyl-3H-indol-1-ium (**1d**)

To a solution of MHI-148 (25.0 mg, 0.04 mmol) in DMF/H_2_O (1:1; 1.00 mL), *N*ε-acetyl-L-lysine (6.96 mg, 0.04 mmol) and ^i^Pr_2_NEt (6.27 μL, 0.04 mmol) were added and the reaction was stirred at 60 °C monitored by Agilent LC-MS. The reaction reached equilibrium after 20 h and the solvent was removed under a stream of nitrogen gas and purified by preparative reversed-phase HPLC (10%–95% CH_3_CN/water containing 0.05% TFA). Compound was lyophilized to obtain blue solid (4.33 mg, 18%). ^1^H-NMR (400 MHz, DMSO) δ 8.02 (d, *J* = 9.4 Hz, 1H), 7.78 (d, *J* = 12.9 Hz, 2H), 7.45 (d, *J* = 7.2 Hz, 2H), 7.35–7.28 (m, 2H), 7.24 (dd, *J* = 11.6, 3.9 Hz, 1H), 7.17 (d, *J* = 8.0 Hz, 2H), 7.09 (t, *J* = 7.4 Hz, 2H), 7.05–7.00 (m, 1H), 5.85 (d, *J* = 13.2 Hz, 2H), 4.43 (dd, *J* = 13.9, 8.8 Hz, 1H), 4.00–3.95 (m, 4jH), 3.68–3.61 (m, 4H), 3.01 (t, *J* = 5.4 Hz, 2H), 2.56 (dd, *J* = 13.9, 7.2 Hz, 2H), 2.42–2.33 (m, 2H), 2.20 (dd, *J* = 13.4, 6.3 Hz, 4H), 1.75 (s, 3H), 1.72–1.63 (m, 6H), 1.60 (s, 12H), 1.52 (dd, *J* = 14.8, 7.4 Hz, 6H), 1.43–1.34 (m, 8H). ^13^C-NMR (100 MHz, DMSO) δ 174.22, 173.25, 168.88, 167.91, 142.71, 140.05, 128.12, 127.58, 122.74, 122.42, 121.96, 120.58, 109.39, 108.49, 95.36, 47.32, 43.24, 42.35, 38.21, 33.47, 28.99, 28.01, 27.60, 25.94, 25.77, 25.62, 24.45, 24.18, 24.11, 24.05, 22.53. HRMS calculated for C_50_H_67_N_4_O_7_^+^ (M)^+^: 835.5004; found 835.4972.

### 3.3. UV-Vis and Fluorescence Analysis

Here, 6 μM of compounds **1a**–**d** samples in 10 mM PBS buffer was prepared by diluting their corresponding stock solution (20 mM) in DMSO using pH 7.24 10 mM PBS buffer. The absorbance and fluorescence of these samples were analyzed using a Varian Cary 100 UV-Vis spectrometer and Varian Cary Eclipse fluorescence spectrophotometer, respectively. The excitation wavelength for compounds **1a**–**d** was set as 740, 720, 580, and 650 nm, respectively. The normalized absorbance and fluorescence data were plotted using GraphPad Prism version 6.0 (GraphPad Software).

### 3.4. NIR Gel Image Protocol

Different cyanines (10 μM; 20 mM stock in DMSO) were incubated with vimentin (1 μM; 1 μg) in pH 7.24 HEPES buffer (50 mM) at 37 °C monitored up to 24 h. For this, 100 ng of each cyanine-vimentin conjugate samples was treated under reducing condition with heating at 95 °C for 10 min and loaded into 15% SDS-PAGE for electrophoresis. The gel was washed with deionized water (10 min × 3 times), and the gel was analyzed by an Odyssey imager to detect the NIR fluorescence.

### 3.5. Preparation of Thiol-Blocked Vimentin

Using pH 7.24 HEPES buffer (50 mM), thiol-blocked vimentin was prepared by incubating 6-maleimide-hexanoic acid (6-MA, 10 μM) with vimentin (1 μg, 1 μM) for 18 h at 37 °C. The thiol-blocked vimentin solution was directly used to incubate with MHI-148 (1 μM) without further purifications. 

### 3.6. Kinetic Study of MHI-148 with Amino Acids in Aqueous Buffer

To a solution of MHI-148 (400 μM) in pH 8.00 HEPES buffer (500 μL), 100 μL of each amino acid solution (2.00 mM) in pH 8.00 HEPES buffer was added to make a final concentration of 200 μM of each reagent. The reaction was incubated and shaken at 37 °C and was monitored using HPLC at 600 nm from 0 to 27 h to reach reaction equilibrium. The data was plotted using GraphPad Prism version 6.0 (GraphPad Software).

### 3.7. NIR Gel Image of MHI-148 with Different Proteins

Different proteins, including NEDD8, Ubc12, truncated suPAR (residues 1-281), NAE, PCSK9, and EGFR (4 μM; 1 μg), were incubated with MHI-148 (4 μM) in pH 7.24 HEPES buffer (50 mM) at 37 °C for 3 h. For this, 500 ng of each protein samples were treated under nonreducing condition with heating at 95 °C for 10 min and loaded into 10% SDS-PAGE for electrophoresis. The gel was washed with deionized water (10 min × 3 times), and the gel was analyzed by an Odyssey imager to detect the NIR fluorescence.

## 4. Conclusions

MHI-148 can label proteins that have free Cys residues such as serum albumin [35] and vimentin. Other Cy-7 dyes containing *meso*-Cl were only used to label vimentin in this work, but it would be unsurprising if they can be used. It seems clear that this methodology could be applied with a high probability of success to conveniently conjugate *meso*-Cl NIR dyes to antibodies, monobodies, and nanobodies to form selective agents for optical imaging in vivo. Traditional conjugation techniques tend to require modification of the dye to include maleimide or succinimide functionality [28,36,37], but the method developed here circumvents that process. 

## Supplementary Materials

The NMR and mass spectrometry of compounds **1a**–**d** and picture of Coomassie blue staining for SDS-PAGE are available online.
