# Supplementary material for: Site-Specific Labeling of Proteins with Near-IR Heptamethine Cyanine Dyes
Source: Molecules. 2018 Nov 7;23(11):2900. doi: 10.3390/molecules23112900 (PMC6278338; doi:10.3390/molecules23112900)
Supplement: Supplementary file 1 [file molecules-23-02900-s001.pdf]

SUPPORTING MATERIALS

Site-Specific Labeling Of Proteins With Near-IR Heptamethine Cyanine Dyes

Chen-Ming Lin, Syed Muhammad Usama and Kevin Burgess*

Department of Chemistry, Texas A & M University, Box 30012, College Station, TX 77842, USA

E-mail: burgess@tamu.edu

Table of Contents

A. 3
^{1}H NMR 3
^{13}C NMR 3
HRMS 4
B. 5
^{1}H NMR 5
^{13}C NMR 5
HRMS 6
C. 7
^{1}H NMR 7
^{13}C NMR 7
HRMS 8
D. 9
^{1}H NMR 9
^{13}C NMR 9
HRMS 10
E. 11

A. Characterization Spectra of Compound 1a

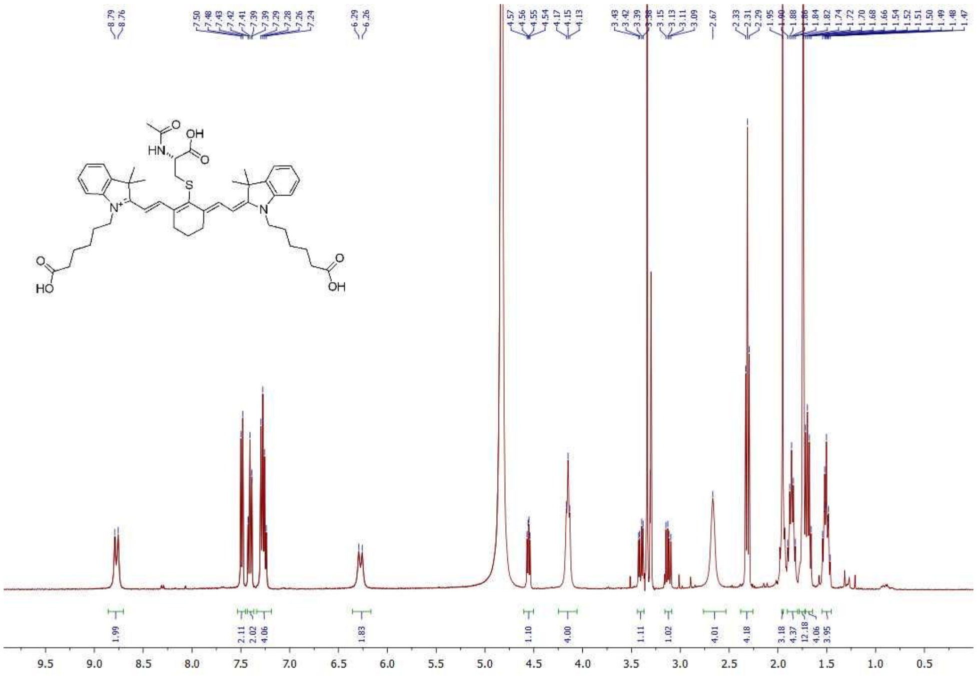

Figure S1. ^{1}H NMR of compound **1a** in Methanol-d4

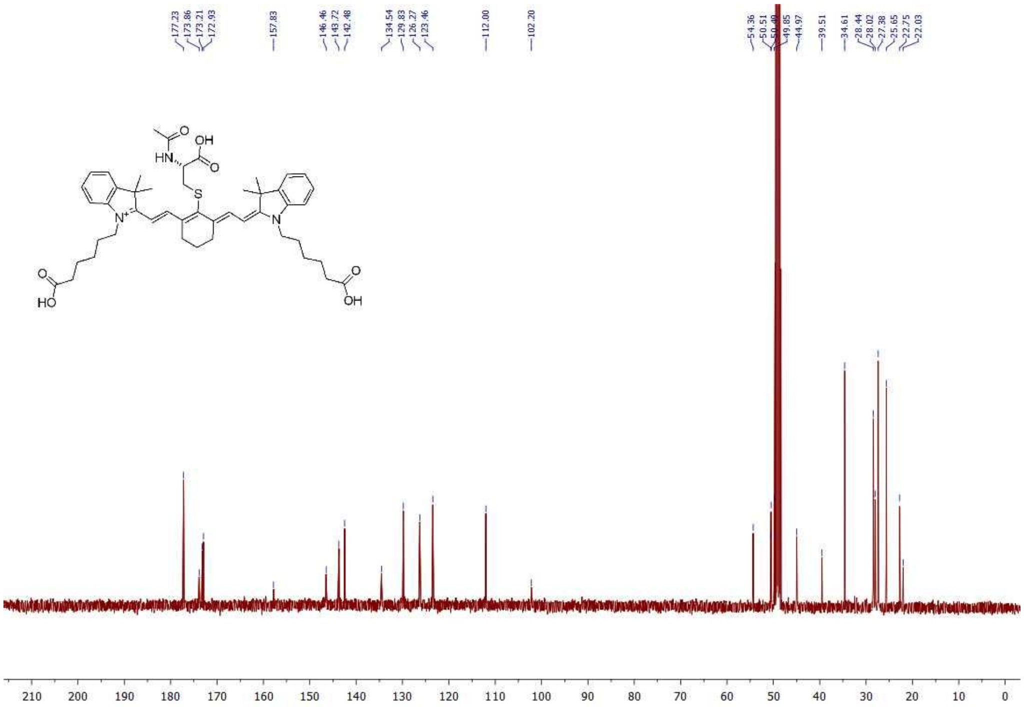

Figure S2. ^{13}C NMR of compound **1a** in Methanol-d4

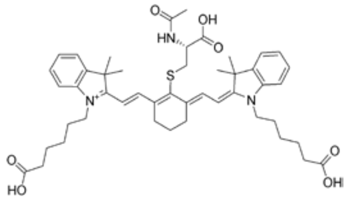

Chemical Formula: $C_{47}H_{60}N_{3}O_{7}S^{+}$
Exact Mass: 810.4146

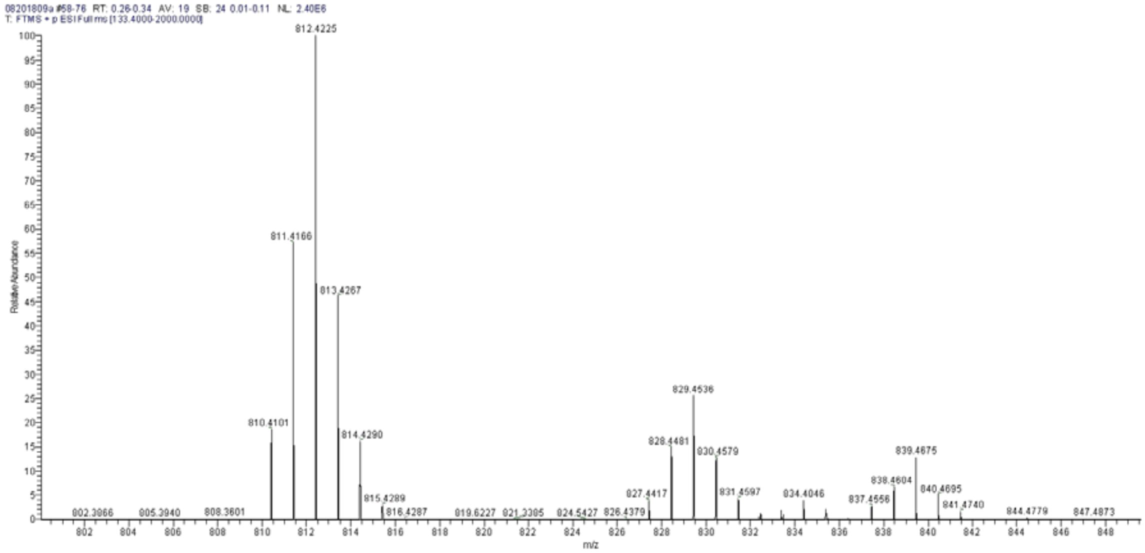

Figure S3. HRMS of compound **1a**

B. Characterization Spectra of Compound 1b

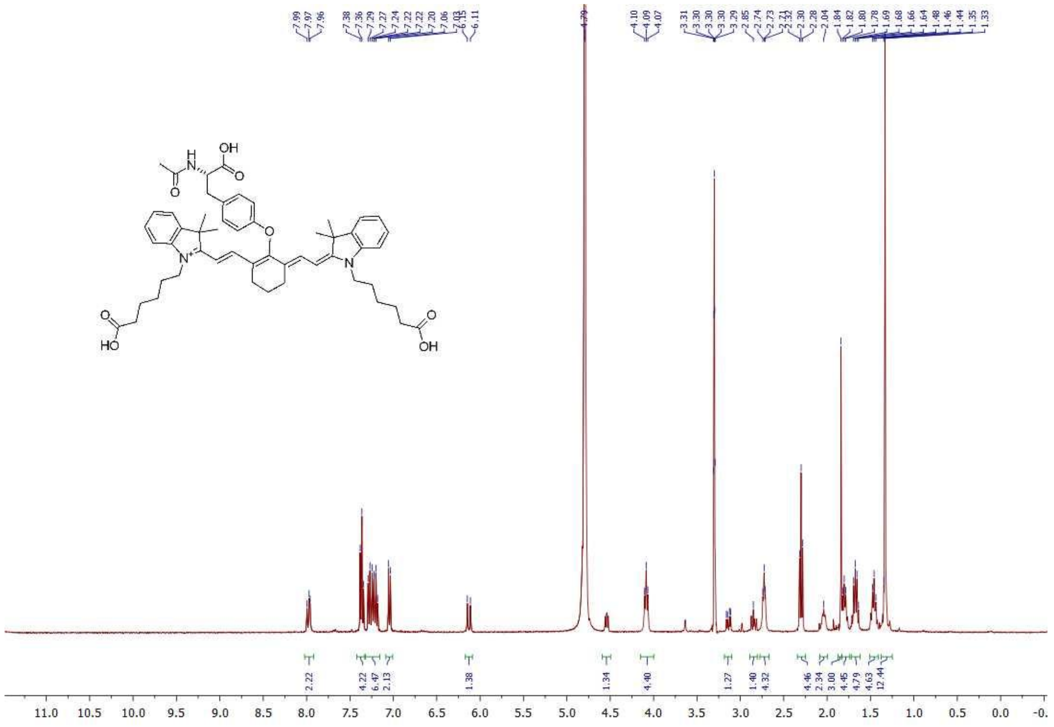

Figure S4. ^{1}H NMR of compound **1b** in Methanol-d4

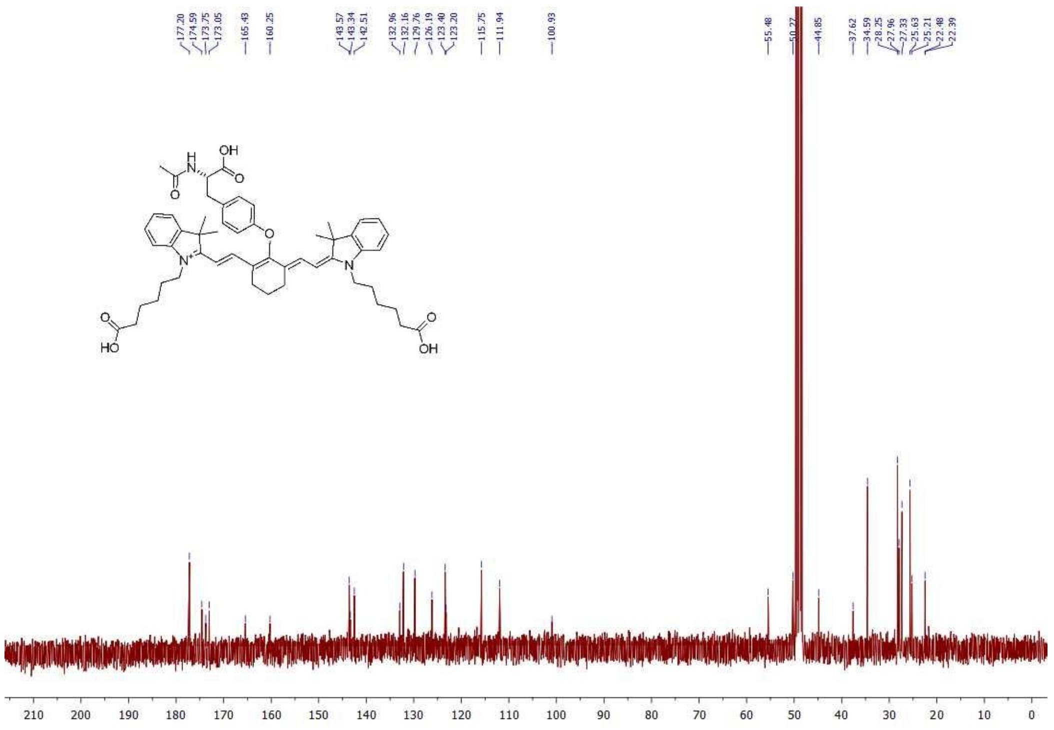

Figure S5. ^{13}C NMR of compound **1b** in Methanol-d4

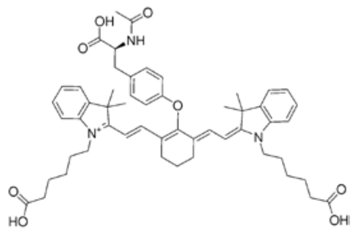

Chemical Formula: $C_{53}H_{64}N_3O_8^+$
Exact Mass: 870.4688

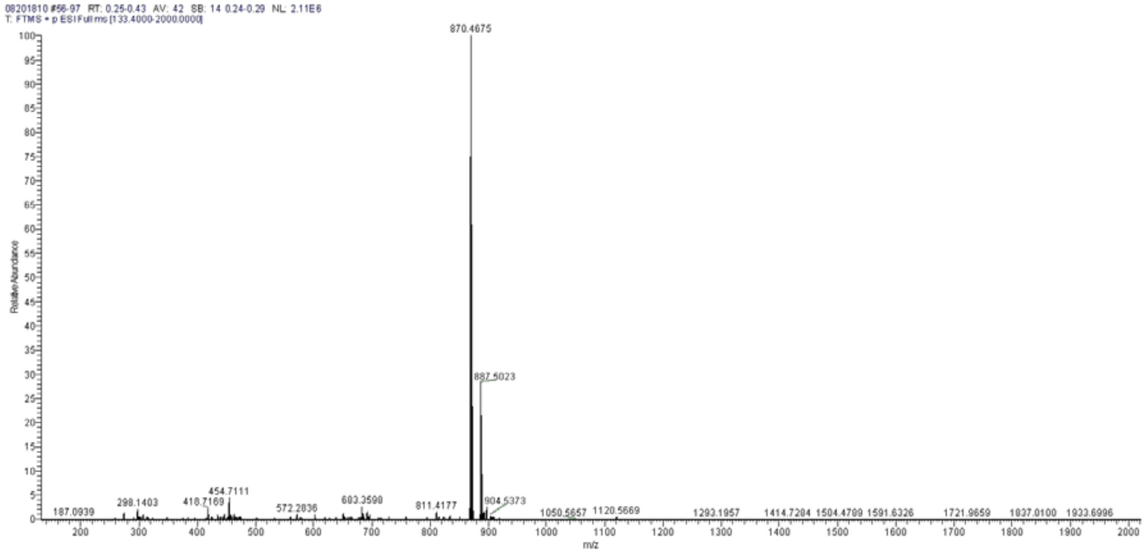

Figure S6. ^{1}H NMR of compound **1b**

C. Characterization Spectra of Compound 1c

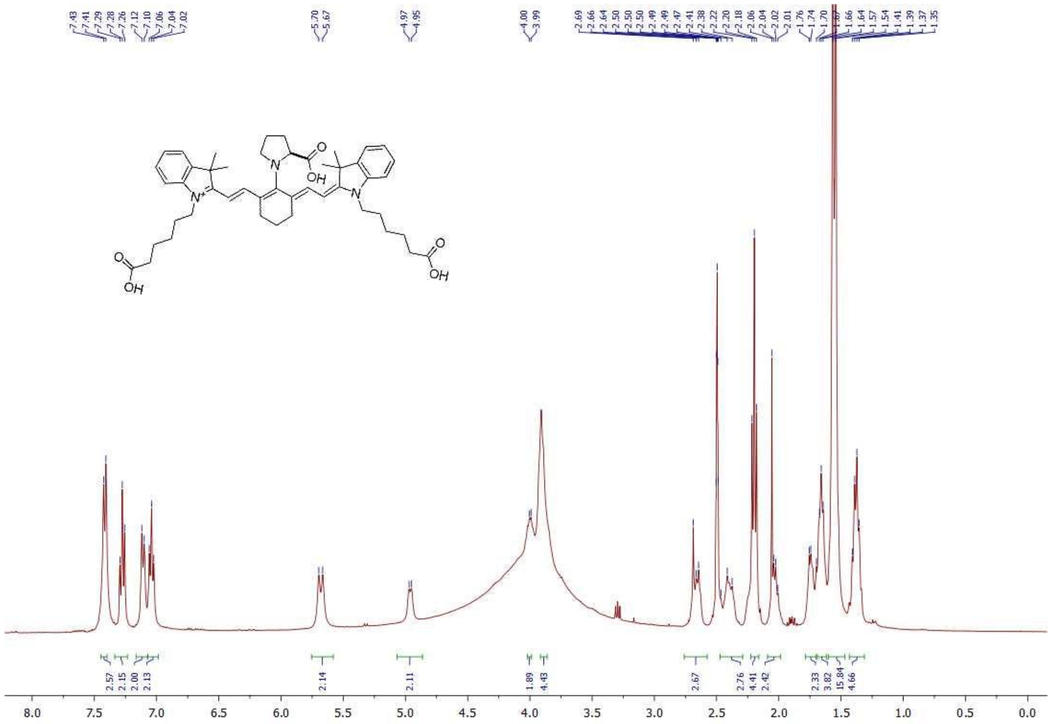

Figure S7. ^{1}H NMR of compound **1c** in Methanol-d4

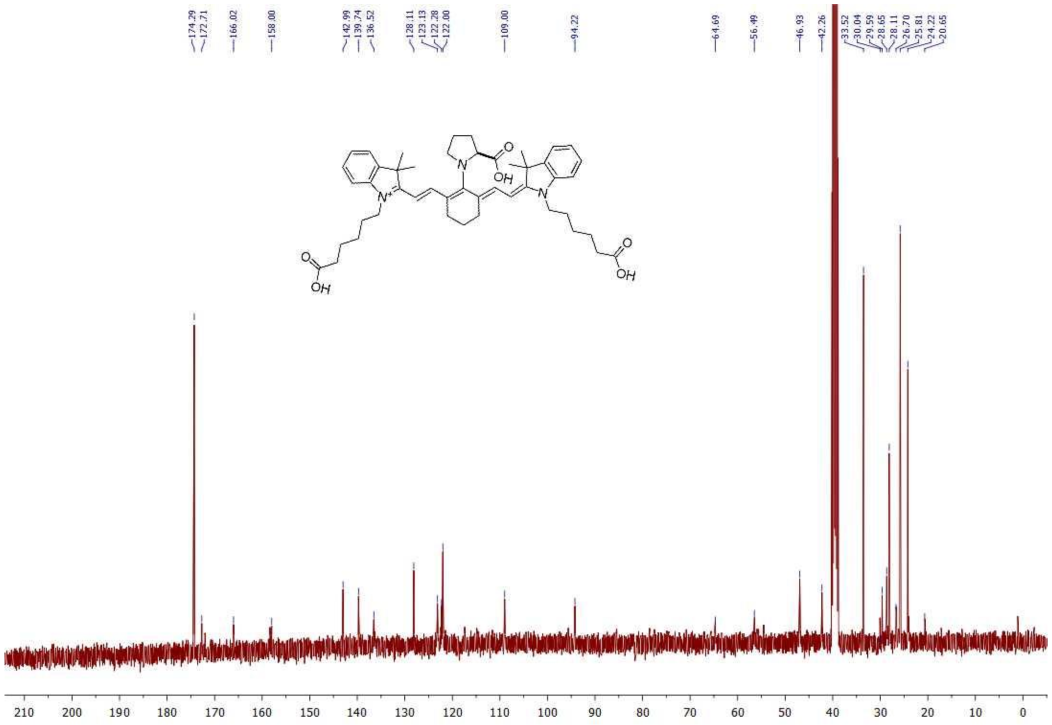

Figure S8. ^{13}C NMR of compound **1c** in Methanol-d4

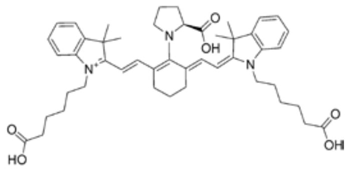

Chemical Formula: $C_{47}H_{60}N_3O_6^+$
Exact Mass: 762.4477

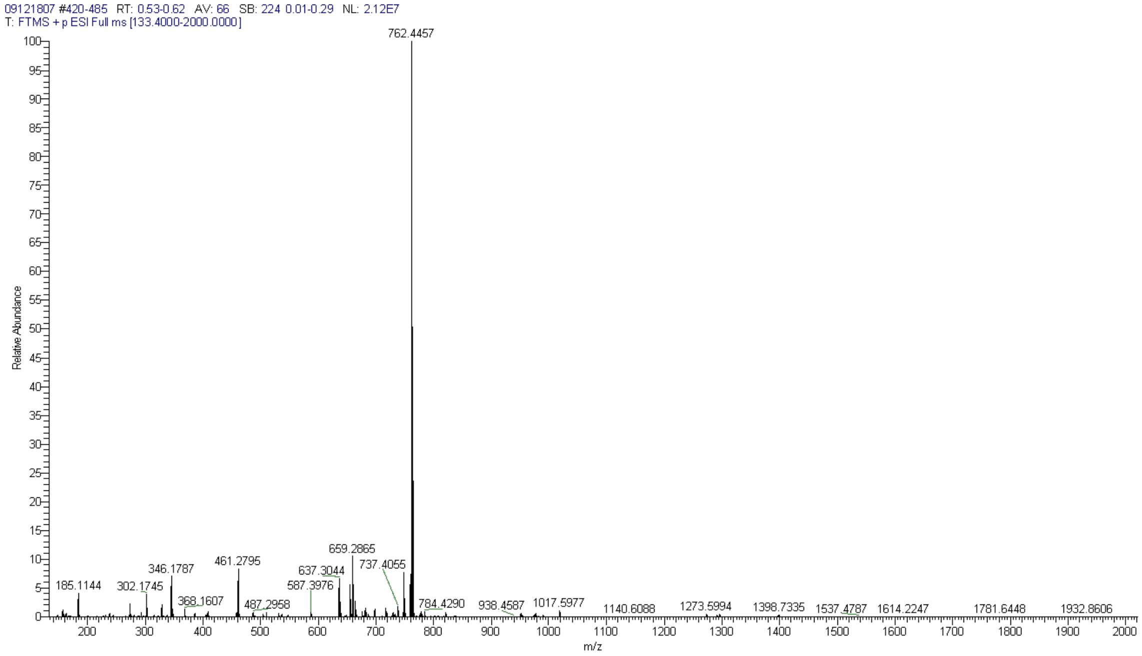

Figure S9. HRMS of compound **1c**

D. Characterization Spectra of Compound 1d

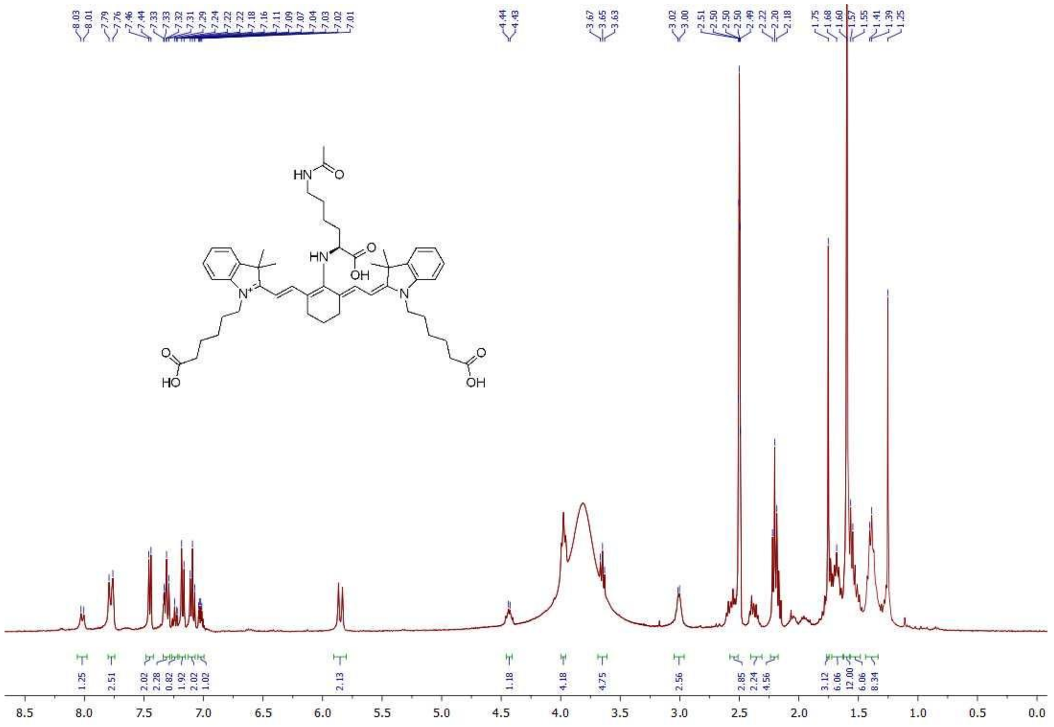

Figure S10. ^{1}H NMR of compound **1d** in Methanol-d4

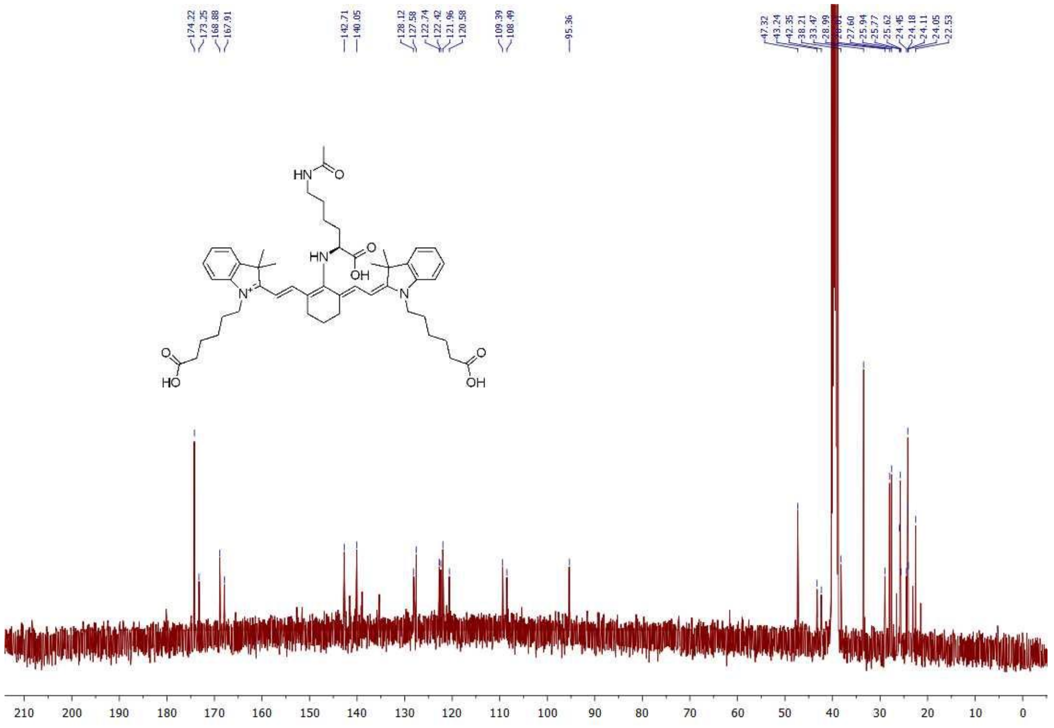

Figure S11. ^{13}C NMR of compound **1d** in Methanol-d4

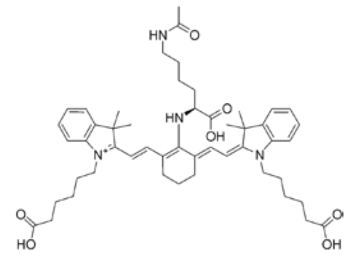

Chemical Formula: $C_{50}H_{67}N_{4}O_{7}^{+}$
Exact Mass: 835.5004

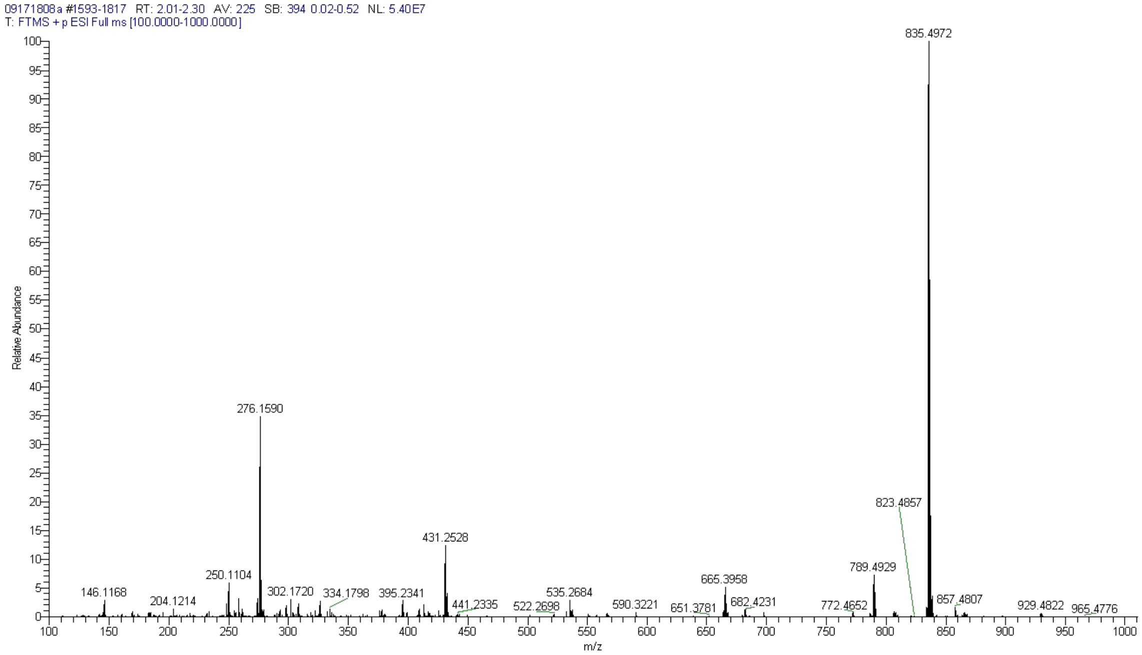

Figure S12. HRMS of compound **1d**

E. Coomassie Blue Staining of Diverse Proteins Gel

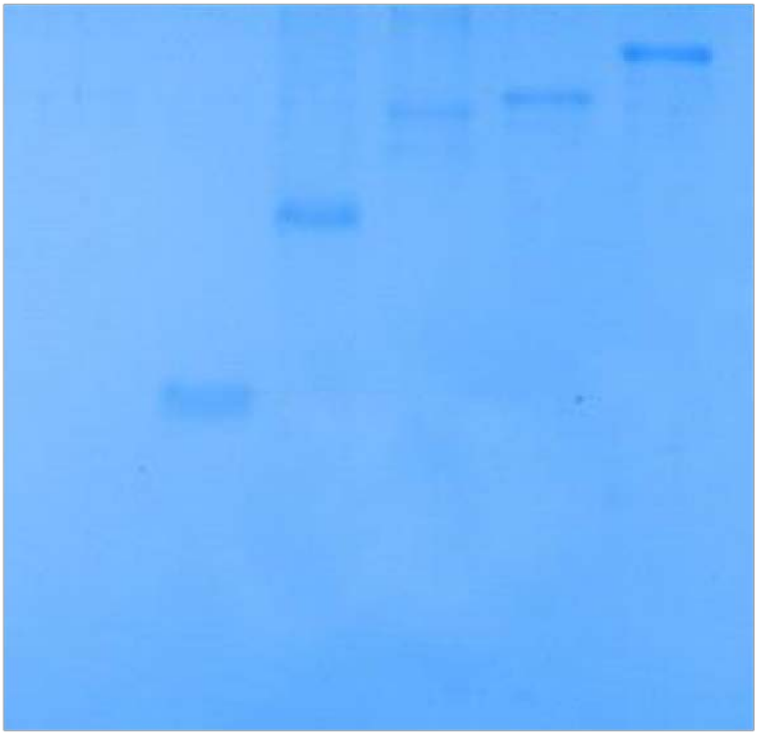

Figure S13. CBB-G250 staining of SDS-PAGE gel referred to Figure 6.
